# Bradycardia associated with remdesivir treatment in coronavirus disease 2019 patients: A propensity score-matched analysis

**DOI:** 10.1097/MD.0000000000044501

**Published:** 2025-10-03

**Authors:** Salman Mohammed, Justin Bauzon, Kavita Batra, Celica Cosme, Kim Inciong, Elli Tian, Fadi Azar, Kevin Lee, Nazanin Houshmand, Uyen Pham, Ariyon Schreiber, Aditi Singh

**Affiliations:** a Kirk Kerkorian School of Medicine at the University of Nevada, Las Vegas (UNLV), NV; b Department of General Surgery, Digestive Disease Institute, Cleveland Clinic Foundation, Cleveland, OH; c Department of Medical Education and Office of Research, Kirk Kerkorian School of Medicine at UNLV, Las Vegas, NV; d Department of Internal Medicine, Kirk Kerkorian School of Medicine at UNLV, Las Vegas, NV.

**Keywords:** bradycardia, propensity score-matched analysis, remdesivir

## Abstract

Remdesivir is an antiviral drug recommended for the treatment of COVID-19. The therapeutic benefits of remdesivir remain under investigation, with further literature emerging regarding its cardiac side-effect profile. The objective of this study was to investigate the association of remdesivir with bradycardia using a propensity score-matched analysis. Secondary outcomes included assessing in-patient mortality and hospital length of stay following remdesivir treatment for COVID-19. A single-institution retrospective study investigating treatment with and without remdesivir in 645 patients admitted for COVID-19 management was performed. Following treatment with remdesivir, patients were analyzed for episodes of bradycardia, defined in this study as 2 consecutive episodes of a heart rate <60 beats per minute. Matched cohorts were generated after controlling for potential confounders associated with remdesivir exposure. Subsequently, a propensity score-matched analysis was conducted to minimize selection bias and to account for the characteristics between the 2 comparison groups. In a sample of 645 COVID-19 patients, 58% were in the treatment group (Remdesivir+) and 42% were in the control group (Remdesivir-). 59% were males with a mean age of 57 ± 14.5 years. After propensity score matching, the remdesivir + group had a higher incidence of bradycardia (48.5% vs 30.7%, *P* = .028). The remdesivir + group was found to have a lower rate of in-patient mortality (1.0% vs 3.0% *P* < .001) and longer in-patient length of stay (13.7 vs 6.3 days, *P* < .001) compared to the remdesivir- group. Patients with COVID-19 had a greater prevalence of bradycardia following treatment with remdesivir. For these patients, remdesivir reduced in-patient mortality compared with untreated COVID-19 patients.

## 1. Introduction

In May 2020, remdesivir was approved under Emergency Authorization Use by the US Food and Drug Administration for the treatment of Coronavirus 2019 disease (COVID-19) after the Adaptive COVID-19 Treatment Trial (ACTT-1) demonstrated a significant decrease in recovery time in patients treated with remdesivir compared to a placebo group.^[1]^ As of April 2023, the National Institutes of Health recommends the administration of remdesivir to all in-hospital patients requiring minimal oxygen support or for those who meet criteria for severe COVID-19 infection.^[2]^ Despite this current recommendation, literature since the ACTT-1 trial has been mixed concerning remdesivir’s purported treatment benefits, making remdesivir’s reduction in mortality debatable today.^[3,4]^

Following the widespread use of remdesivir for the treatment of COVID-19, bradycardia emerged as an increasingly reported side-effect of undetermined clinical significance.^[5,6]^ To date, explanations on whether bradycardia develops from remdesivir are mostly limited to observational data and case studies.^[5,7]^ Simultaneously, the underlying cardiac manifestations of COVID-19 as a cause of bradycardia cannot be fully ruled out.^[5,7]^ In theory, remdesivir may decrease electrical conduction through the atrioventricular (AV) node via a mechanism similar to adenosine, resulting in bradyarrhythmia.^[8]^ Earlier literature has suggested that remdesivir-associated bradycardia may confer an up to 6-fold increased risk of mortality,^[9–11]^ but recent studies have challenged these clinically relevant outcomes.^[12,13]^ Earlier literature regarding hospital length of stay (LOS) has also varied, from reducing hospital admission length by 5 days to increasing it by one.^[1,4]^

Given that most of the previous studies investigating remdesivir-associated bradycardia are observational in nature and are inherently limited by selection bias, the present study aims to mitigate this bias by investigating the incidence of bradycardia in patients treated with remdesivir relative to those not treated with remdesivir for COVID-19 disease in a propensity-matched sample. Additionally, we compare the clinical outcomes of in-patient mortality and hospital LOS in patients with COVID-19 treated with and without remdesivir. We hypothesize that treatment with remdesivir presents a clinically significant association with bradycardia that may influence patient mortality and LOS, the results of which would strengthen our understanding of remdesivir as a mainstay treatment for COVID-19.

## 2. Materials and methods

### 2.1. Study design and setting

A retrospective analysis of 645 consecutive patients at University Medical Center of Southern Nevada who were admitted for COVID-19 infection as defined by the National Institute of Health’s COVID-19 treatment guidelines via polymerase chain reaction were identified between March 1, 2020 to March 31, 2021.^[2]^

### 2.2. Eligibility criteria

All study subjects were screened for COVID-positivity prior to admission. Patients were excluded if they were <18 years old, were pregnant, had a less than 2-day LOS, had evidence of severe hepatic dysfunction (AST or ALT > 5 times the upper limit of normal), demonstrated renal impairment (eGFR < 30 mL/min), or received atrioventricular nodal (AVN) agents, such as beta-blockers, calcium channel blockers (e.g. diltiazem, verapamil), and other anti-arrhythmic agents during hospital admission.^[14]^ Two physician reviewers independently verified all inclusion criteria.

Patients were assigned to 1 of 2 cohorts based on whether they received remdesivir treatment on admission for COVID-19: treatment group (Remdesivir+) or control group (Remdesivir-). All remdesivir + patients received a full 5-day course of remdesivir (200-mg intravenous [IV] loading dose, 4-day 100-mg IV daily maintenance dose).^[14]^ All remdesivir- patients did not receive any antivirals in the context of this study. A total of 645 patients (375 treatment vs 270 control) were included in the final analysis.

### 2.3. Ethics approval

This study was conducted following University Medical Center of Southern Nevada institutional review board (IRB) approval (UMC IRB #: UMC-2021-362). This research study was conducted retrospectively from data obtained for clinical purposes. We consulted extensively with the IRB of the University Medical Center of Southern Nevada, who determined that our study did not need ethical approval. An IRB official waiver of ethical approval was granted from the University Medical Center of Southern Nevada.

### 2.4. Variables and measures

Independent variables included demographics (age, race/ethnicity, sex, body mass index [BMI]), medical history (history of arrhythmia, thyroid disease, sleep apnea), vitals obtained on admission (HR, temperature, oxygen saturation), highest level of supplemental oxygenation administered (i.e. endotracheal intubation, bilevel positive airway pressure [BiPAP]), and tobacco use. Outcomes assessed were presence of bradycardia, mortality, and LOS.

Specific to our institution and studies referenced, sinus bradycardia was defined as a heart rate (HR) <60 beats per minute.^[15]^ For the purposes of this study, an episode of bradycardia was defined as 2 consecutive episodes of sustained HR <60 bpm at least 4 hours apart. In the Remdesivir + cohort, episodes of bradycardia were monitored from initiation of remdesivir up to 5 days after completion of a full treatment course. This therapeutic window was based on remdesivir’s half-life of 20 to 27 hours, or when approximately 95% of the active metabolite would be expected to be eliminated.^[6,15]^ The Remdesivir- group was evaluated for bradycardia from hospital admission to 10 days after admittance. Each patient’s HR was monitored through various modalities such as electrocardiogram, telemetry or pulse oximeter, based on the level of care the patient received.

The matched cohorts were generated using variables expected to serve as potential confounders associated with remdesivir exposure: age, gender, race or ethnicity, hyperlipidemia, and modality of oxygenation. Dyslipidemia has been shown to serve as an independent predictor of in-patient mortality in remdesivir-treated patients.^[6,16–18]^ Mechanical ventilation status has also been associated with COVID-related bradycardia,^[6,19]^ which may be secondary to the sedative and narcotic agents given to intubated patients that have known negative chronotropic effects.^[20,21]^

### 2.5. Data collection

Clinical information was obtained from an electronic record system via a team of medical trainees using a combination of semiautomated procedures as well as manual extraction.^[22]^ Data cleaning was performed to remove implausible values (example: HR of 600 as a misinput, or HR < 20 or > 200 based on physiologic norms and clinical judgement) and transcription errors.^[23]^

### 2.6. Sample size justification

A formal a priori power analysis was conducted using G*Power software (version 3.1) to determine the minimum required sample size for our analyses, given the limited prior literature on this specific research question. Standard conventions for effect sizes were applied: Cohen d = 0.5 for *t*-tests, and w = 0.3 for chi-square tests, as recommended in the literature (Added 21 cohen J). Based on a power level (1-β) of 0.95 and an alpha of 0.05, the estimated sample sizes required were 210 for *t*-tests and 220 for chi-square tests. To ensure sufficient power across all planned analyses, we adopted the highest of these estimates (N = 220) as the benchmark. Our final analytic sample of 645 patients far exceeded this threshold, offering robust statistical power and enabling meaningful subgroup comparisons within the propensity score-matched (PSM) design. Moreover, large retrospective cohort studies such as ours benefit from enhanced power due to real-world data volume. Prior literature supports that sample sizes exceeding 200 per group can reliably detect moderate effects in propensity score- matched designs.^[24,25]^ Our findings are consistent with effect estimates from other published studies that examined bradycardia and mortality associated with remdesivir (e.g.,.^[12,13,26]^

### 2.7. Statistical analysis

**Figure 1.**
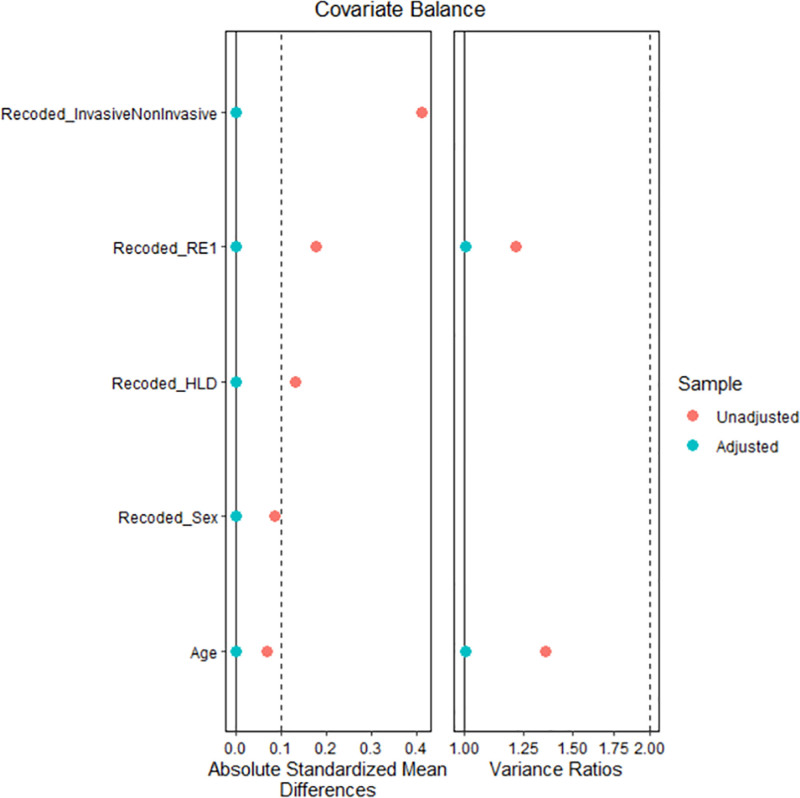
Love plot depicting matching of groups for subsequent PSM analysis. PSM = propensity score-matched

PSM analysis was conducted to minimize selection bias and to account for the characteristics of the 2 comparison groups. The 2 groups were matched on a set of variables, including age, race/ethnicity, gender, hyperlipidemia, type of oxygen supplementation (invasive or noninvasive). Propensity matching was conducted through “Matchit” and “Tableone” packages in the R programming software. A logistic regression was fit to estimate propensity scores (predicted probabilities) for each subject.^[27]^ As an intermediary step before matching, the distribution of propensity scores across 2 groups was assessed through visual inspection of love plots (Fig. 1). Two groups were matched using the exact matching algorithm. The balance of covariates was assessed through the standardized mean differences and variance ratios. The standardized mean differences values below 0.1 and variance ratios close to 1 were considered optimal for an adequate covariate balance.^[28–31]^ Continuous data were reported as means and standard deviations (S.D.), while categorical data were reported as frequencies and proportions (%). For outcome analyses in a post-match sample, McNemar tests (with continuity correction) and paired *t*-tests were conducted to compare categorical and continuous data, respectively. The significance level was set at .05. All analyses were conducted through R and SPSS version 28 software (Chicago).

## 3. Results

Table 1 summarizes the characteristics of the unmatched cohort. The mean age of the sample was 57.31 ± 14.5 years, with 59% males. In a sample of 645 patients, 58% (N = 375) were in the remdesivir + group and nearly 42% (N = 270) were in the remdesivir- group. Remdesivir- subjects had a significantly larger proportion of Black patients (7.5% in control vs 14.4% in treatment group, *P* = .03) and current smokers (4.3% vs 11.0%, *P* = .002). Conversely, remdesivir + patients had a significantly higher prevalence of type 2 diabetes mellitus compared to the control group (45.2% vs 35.4%, *P* = .01) (Supplemental Digital Content, https://links.lww.com/MD/Q48). Further, there were no statistically significant differences in the mean BMI between both groups. However, analyzing the BMI subcategories showed that those in the remdesivir- cohort had a greater proportion of patients who were underweight (7.5% vs 14.4%, *P* = .03) while those in the remdesivir + group were more obese (15.2% vs 9.3%, *P* = .001).

**Table 1:** Characteristics of the unmatched cohort (N = 645).

Variable name	Categories	Control (N = 270)	Treatment (N = 375)	*P*-value
Age (Mean ± S.D.)	**–**	58.26 ± 15.9	56.63 ± 13.2	.2
Gender, N (%)	Female	121 (44.8)	145 (38.7)	.1
	Male	149 (55.2)	230 (61.3)	
Race/Ethnicity, N (%)*	White	38 (16.1)	71 (19.7)	**.03**
	Black	34 (14.4)	27 (7.5)	
	Hispanic	140 (59.3)	214 (59.4)	
	Others	24 (10.2)	48 (13.3)	
Body mass index categories, N (%)	Underweight (BMI < 18.5)	14 (5.2)	1 (0.3)	**<.001**
	Normal weight (BMI 18.5–24.9)	51 (18.9)	69 (18.4)	
	Overweight (BMI 25–29.9)	82 (30.4)	86 (22.9)	
	Obese Class 1 (BMI 30–34.9)	64 (23.7)	94 (25.1)	
	Obese Class 2 (BMI 35–39.9)	25 (9.3)	57 (15.2)	
	Obese Class 3 (BMI > 39.9)	34 (12.6)	68 (18.1)	
History of tobacco use, N (%)*	Current (0)	29 (11.0)	16 (4.3)	**.002**
	Former (1)	37 (14.0)	41 (10.9)	
	Never (2)	198 (75.0)	318 (84.8)	
Type 2 Diabetes mellitus, N (%)*	Yes	92 (35.4)	169 (45.2)	**.01**
	No	168 (64.6)	205 (54.8)	
Hyperlipidemia, N (%)*	Yes	72 (27.6)	115 (30.7)	.4
	No	189 (72.4)	259 (69.3)	
Prior arrhythmia, N (%)*	Yes	9 (3.4)	5 (1.3)	.08
	No	257 (96.6)	369 (98.7)	

BMI = body mass index.

Bold indicates statistical significance (*P* < .05).

* Some percentages may not add to 100% due to missing information.

The results of the primary outcomes in the unmatched study group are outlined in Table 2. Remdesivir + patients had a higher incidence of bradycardia (48.5% vs 29.6% *P* < .001) and a longer hospital LOS (16.6 vs 8.6 days, *P* < .001). The remdesivir + cohort had a greater rate of in-patient mortality rate (17.1 vs 6.3%, *P* < .001) compared to the remdesivir- group.

**Table 2:** Comparing the primary outcomes among unmatched study groups (N = 645).

Variable name	Control (N = 270)	Treatment (N = 375)	*P*-value
In-patient mortality, N (%)			
Yes	17 (6.3)	64 (17.1)	**<.001**
No	253 (93.7)	311 (82.9)	
Bradycardia, N (%)			
Yes	80 (29.6)	182 (48.5)	**<.001**
No	190 (70.4)	193 (51.5)	
Length of hospital stay (Mean ± SD)	8.61 ± 17.44	16.62 ± 23.25	**<.001**

SD = standard deviation.

Bold indicates statistical significance (*P* < .05).

Following propensity score matching, the treatment group was found to have a higher incidence of bradycardia (48.5% vs 30.7%, *P* = .028) compared to the control group (Table 3). Subjects in the remdesivir + group had a lower rate of in-patient mortality (1.0% vs 3.0% *P* < .001) and longer in-patient LOS (13.7 vs 6.3 days, *P* < .001).

**Table 3:** Primary outcomes among study groups following propensity score matching (N = 202).

Variable name	Control (N = 101)	Treatment (N = 101)	*P*-value
Bradycardia, N (%)	31 (30.7)	49 (48.5)	**.028**
In-patient mortality, N (%)	3 (3.0)	1 (1.0)	**<.001**
Length of hospital stay (Mean ± SD)	6.3 ± 5.5	13.7 ± 12.8	**<.001**

Bold indicates statistical significance (*P* < .05).

## 4. Discussion

Our study evaluated the incidence and clinical implications of bradycardia associated with remdesivir treatment in COVID-19 patients, defined as any episode of bradycardia from initiation of remdesivir up to 5 days after completion of a full treatment course. The major findings of our study are: the remdesivir + group showed a 50% higher incidence of bradycardia compared to the control group (Remdesivir-group), the remdesivir + group showed a 3-fold decrease in mortality compared to the remdesivir- group, and the remdesivir + group had a hospital LOS that was twice as long compared to the control group.

Bradycardia is the most reported cardiovascular side-effect of remdesivir in COVID-19 patients, although its prevalence varies widely, from 3.6% up to 60%.^[32–34]^ This variation may be due to individual patient factors and comorbidities, the severity of COVID-19 infection, or inherent to the observational study designs including selection bias and varied parameters in how bradycardia is defined.^[34,35]^ In a more recent study, a low-normal baseline HR (67–89bpm) before receiving remdesivir was the only significant risk factor for developing remdesivir-associated bradycardia.^[11]^ Recent meta-analyses have demonstrated an increased risk of bradyarrhythmia in COVID-19 patients treated with remdesivir.^[5,36,37]^ In our study, the incidence of remdesivir-associated bradycardia was higher in patients treated with remdesivir compared to those not treated with remdesivir, which is consistent with prior findings.^[11,35–39]^ Especially in relation with Attena et al recent PSM analysis, our study also found bradycardia associated with remdesivir treatment but with an incidence that was twice as great (49% vs 20%). To the authors’ knowledge, this is the strongest supporting evidence to date that bradycardia is intrinsically tied to remdesivir treatment. Such findings warrant increased cardiac monitoring for patients receiving remdesivir, given its uncertain side-effect profile.^[33]^

Although the mechanism behind remdesivir-induced bradycardia is unknown, it is theorized that remdesivir, an antiviral RNA-dependent RNA polymerase inhibitor, acts via its active metabolite, which is an adenosine triphosphate analog.^[40]^ Adenosine is a negative chronotropic and dromotropic agent known to suppress cardiac pacemaker activity at the AV node, which in theory results in bradycardia.^[41]^ This specific adenosine analogue derived from remdesivir has a significantly longer half-life lasting hours, as opposed to adenosine’s half-life lasting up to seconds, which may make the effects of remdesivir observable via cardiac monitoring devices.^[42]^ Another proposed mechanism is explained by adenosine’s parasympathomimetic effect that increases vagal activity, thereby inducing bradycardia.^[43]^ Other studies have also studied the risk of a prolonged QT interval following treatment with remdesivir.^[43–45]^

It is worth exploring COVID-19 infection itself as a potential cause of bradyarrhythmia. Recent data has suggested that the bradycardia observed in COVID-19 patients may be a result of the disease course itself and that remdesivir may instead be a confounding variable.^[12]^ The exact mechanisms are not fully understood, but bradycardia secondary to COVID-19 may be a multifactorial result of the inflammatory responses to the SARS-CoV-2 infection resulting in acidosis, electrolyte abnormalities, hypoxemia, direct effects of inflammatory cytokines, and sinoatrial node involvement.^[7,46]^ Previous observational studies have supported the hypothesis that bradycardia is a result of COVID-19.^[9,10,47]^ Umeh et al found higher rates of mortality in bradycardic COVID-19 patients despite showing no relationship between remdesivir and bradycardia. A meta-analysis of 59 patients from case reports and case series found an increased incidence of bradycardia with severe or critical COVID-19 infection; over half of these cases were found to have bradycardia directly associated with the disease course with only a small fraction (8.5% of patients) having utilized remdesivir treatment.^[48]^

Since the ACTT-1 trials report of decreased recovery time with use of remdesivir, recent data has emerged questioning remdesivir’s mortality and LOS benefits.^[1]^ In the World Health Organization’s (WHO) Solidarity Trial Consortium, remdesivir demonstrated no improvement in mortality and instead found a 1-day increase in-hospital LOS.^[4]^ This is also consistent with Ohl et al’s study, which analyzed the United States veteran population, and found no improvement in mortality and a 3-day longer LOS. Similarly, Shaikh et al found no improvement in mortality and a 2-day longer LOS. WHO’s randomized trial adjusted for variables not accounted in the ACTT-1 trial, such as patients with good prognosis in the remdesivir arm, which may have skewed remdesivir’s overall positive clinical outcomes.^[4]^ The most recent systematic review to date analyzed 9 RCTs, which showed that remdesivir had no significant effect on all-cause mortality in COVID-19 patients.^[3]^ Additionally, while remdesivir may improve the clinical course of COVID-19 patients resulting in slightly earlier in-patient discharge, this evidence was determined to be weak.^[3]^ Our study supports the notion that remdesivir decreases rates of mortality, although it should be noted that the absolute risk was low (3 in the remdesivir- group vs 1 in the remdesivir + group). This is concordant with other propensity score analyses using large databases where survival benefits range from 17%^[49]^ to 33%.^[50]^ Two recent studies using the propensity score analysis found a mortality reduction rate of up to 50% and 82%, along with other clinical benefits, such as decreased intubation.^[51,52]^ With the propensity score design, however, a relationship between remdesivir and mortality is not always evident.^[53]^ Other retrospective studies emulating aspects of the RCT design also concluded that remdesivir may decrease mortality.^[26,53]^

To our knowledge, this is among the first PSM analyses to investigate remdesivir-associated bradycardia that directly addresses issues with selection bias.^[13]^ A similar study from Attena et al using PSM analysis revealed similar findings, such as reduced mortality, and increased incidence of bradycardia following remdesivir treatment.^[13]^ Otherwise, previous data has mostly been limited to case descriptions and observational studies, with 3 meta-analyses studying remdesivir-induced bradycardia published to date.^[5,36,37,42,54]^ Remdesivir has shown to be an effective treatment and remains the only Food and Drug Administration-approved antiviral treatment for COVID-19 patients, which makes randomized trials comparing a non-remdesivir trial arm not feasible. A PSM analysis minimizes potential biases in observational studies - selection bias, non-randomization, unaccounted confounders - and presents, at least, an estimate of causal effect by matching patients among study cohorts using selected clinical features.^[55]^ In the present study, matched characteristics included demographics (age, sex, race/ethnicity), method of oxygenation, and hyperlipidemia - features observed to be differentiated among study groups in a previous analysis^[6]^ (Supplemental Digital Content, https://links.lww.com/MD/Q48). Our analysis offers one of the strongest supporting data regarding bradycardia associated with remdesivir treatment.

Several limitations to this study exist. Although a propensity score match analysis was performed in order to minimize these confounding variables, this method cannot supersede the results of a randomized controlled trial performed on the same subject matter. In fact, unmeasured covariates, such as chronic lung and cardiovascular disease, concurrent organ dysfunction, severity of disease and, immunocompromised status among other factors may still contribute to residual confounding bias.^[11,56,57]^ Our sample consisted of patients with severe COVID-19 infection, which is a condition associated with bradycardia itself, regardless of remdesivir treatment. Potentially, other conditions associated with bradycardia, such as during sleep or rest, may also present difficulties in measuring remdesivir-associated bradycardia.^[16]^ A subgroup analysis analyzing our sample according to the severity of bradycardia and its subtypes was also not performed. Our single-center setting may also represent a sampling weakness. However, the relatively large sample size, propensity score matching method of analysis, and fairly diverse and equivalent representation of patient demographics may strengthen our study. Further randomized studies are needed to explore the mechanisms of bradycardia associated with remdesivir treatment and understand its effects on mortality and other clinical characteristics.

## 5. Conclusion

Remdesivir treatment for hospitalized COVID-19 patients may be associated with a higher incidence of bradycardia, lower in-patient mortality, and longer hospital LOS. Whether bradycardia associated with remdesivir treatment is of clinical significance remains under question. Further investigation is needed to understand the clinical outcomes of remdesivir with the implications of bradycardia associated with remdesivir in mind.

## Author contributions

**Conceptualization:** Salman Mohammed, Justin Bauzon, Kavita Batra, Ariyon Schreiber, Aditi Singh.

**Data curation:** Justin Bauzon, Kavita Batra, Elli Tian, Fadi Azar, Kevin Lee, Nazanin Houshmand, Uyen Pham.

**Formal analysis:** Kavita Batra.

**Investigation:** Salman Mohammed, Justin Bauzon, Kavita Batra, Ariyon Schreiber.

**Methodology:** Justin Bauzon, Kavita Batra, Ariyon Schreiber.

**Project administration:** Justin Bauzon.

**Software:** Kavita Batra.

**Supervision:** Salman Mohammed, Justin Bauzon, Aditi Singh.

**Validation:** Salman Mohammed, Kavita Batra.

**Writing – review & editing:** Salman Mohammed, Justin Bauzon, Celica Cosme, Kim Inciong, Elli Tian, Fadi Azar, Kevin Lee, Nazanin Houshmand, Uyen Pham, Ariyon Schreiber, Aditi Singh.

**Writing – original draft:** Salman Mohammed, Justin Bauzon, Kavita Batra, Celica Cosme, Kim Inciong, Aditi Singh.

## Supplementary Material
