# Supplementary material for: Bradycardia associated with remdesivir treatment in coronavirus disease 2019 patients: A propensity score-matched analysis
Source: Medicine (Baltimore). 2025 Oct 3;104(40):e44501. doi: 10.1097/MD.0000000000044501 (PMC12499812; doi:10.1097/MD.0000000000044501)
Supplement: Supplementary file 1 [file medi-104-e44501-s001.docx]

**(Supplemental Content) Table: Cardiac, Laboratory and Vital Sign parameters**

Compared to the control group, patients in the treatment group had a lower minimum (51.3 vs. 58.0, p<0.001) and maximum heart rate (106.6vs. 110.8, p = 0.01). The RDV+ group also had lower baseline oxygen saturation recorded (82.6 vs 86.7 p<0.001) compared to the control group.

Patients in the treatment group also had higher rates of oxygenation requirements during hospital stay. The use of high-flow nasal cannula (HFNC) and prevalence of intubation were higher in those treated with remdesivir (135 vs 22 patients, p<0.001 and 77 vs 14 patients, p<0.001, respectively). In contrast, patients who did not receive treatment required less invasive supplemental oxygen support such as nasal cannula (NC) (157 vs. 192, p<0.001).

*(Supplemental Content) Table. Cardiac, laboratory and other vital characteristics of study population (N=645)*

| Variable | Control | Treatment | P value |
| --- | --- | --- | --- |
| **Vital Signs** |  |  |  |
| Lowest HR (bpm) | 57.96±13.1 | 51.32±11.8 | **<0.001** |
| Highest HR (bpm) | 106.62±19.7 | 110.78±20.7 | 0.01 |
| Minimum Temperature (Celsius) | 37.3±0.32 | 35.7±0.57 | **<0.001** |
| Maximum Temperature (Celsius) | 37.8±1.27 | 37.8±0.89 | 0.7 |
| Minimum SpO2 (mm of Hg) | 86.7±12.0 | 82.6±11.5 | **<0.001** |
| Maximum SpO2 (mm of Hg) | 99.02±2.1 | 99.26±1.07 | 0.05 |
| **Highest level of Oxygen required** |  |  |  |
| NC | 192 (71.1) | 157 (41.9) | **<0.001** |
| HFNC | 22 (8.1) | 135 (36.0) |  |
| BiPap | 3 (1.1) | 2 (0.5) |  |
| Intubation | 14 (5.2) | 77 (20.5) |  |
| None | 39 (14.4) | 4 (1.1) |  |
